# The Reliability of Anamnestic Data in the Management of *Clostridium Tetani* Infection in Elderly

**DOI:** 10.3389/fmed.2021.684594

**Published:** 2021-10-28

**Authors:** Gabriele Savioli, Iride Francesca Ceresa, Mauro Giordano, Ilaria Ferrari, Angelica Varesi, Valentina Floris, Ciro Esposito, Barbara Croesi, Giovanni Ricevuti, Monica Calvi, Maria Antonietta Bressan, Enrico Oddone

**Affiliations:** ^1^Emergency Department, Istituto di Ricovero e Cura a Carattere Scientifico (IRCCS) Policlinico San Matteo, Pavia, Italy; ^2^Ph.D University of Pavia, Pavia, Italy; ^3^Internal Medicine, University of Campania “L. Vanvitelli”, Naples, Italy; ^4^Department of Biology and Biotechnology, University of Pavia, Pavia, Italy; ^5^Department of Internal Medicine and Therapeutics, University of Pavia, Pavia, Italy; ^6^University of Pavia Department of Internal Medicine and Maugeri Unit of Nephrology and Dialysis, ICS Maugeri, Pavia, Italy; ^7^Pharmacy, Istituto di Ricovero e Cura a Carattere Scientifico (IRCCS) Policlinico San Matteo, Pavia, Italy; ^8^School of Pharmacy, Department of Drug Sciences, University of Pavia, Pavia, Italy; ^9^Department of Public Health, Experimental, University of Pavia, Pavia, Italy

**Keywords:** tetanus, vaccination, immunity, emergency department, emergency room, risk management, tetanos quick stick

## Abstract

**Background:** Tetanus infection remains a significant complication of wounds. Because most tetanus treatment guidelines rely on anamnestic data collected directly from patients, the congruence between anamnesis and laboratory evidence must be verified, especially in the elderly population.

**Aim:** Assess, in both the geriatric population (>65) and the non-geriatric one, the reliability of anamnestic data for managing patients with tetanus-risk wounds, identified categories of populations most exposed to non-vaccination coverage, and assessed the agreement of the Tetanos Quick Stick (TQS) results with the therapy performed (administration of tetanus vaccine or immunoglobulin).

**Methods:** In this retrospective single-center observational study, patients were asked their immunization status against tetanus vaccination. The decision to administer a vaccine or immunoglobulin was therefore clinical and based on anamnestic criteria. The TQS test was then given to patients who were unaware of their immunity status. Patients who thought they knew it but were not sure were given the TQS test to determine whether the anamnestic collection was supported by the test. The TQS test results were compared with the anamnestic data.

**Results:** Most patients, geriatric and not geriatric, did not know their immune status. Among those who reported knowing their immune status, there was no agreement between the vaccine coverage declared by patients and the TQS test results (*p* < 0.001), mainly in geriatric patients but also in the control group. Elderly and women had significantly lower positive TQS test results (*p* < 0.001). There was a statistically significant discrepancy (*p* < 0.001) between the therapy based on anamnestic data and the TQS test results.

**Conclusion:** The reliability of anamnestic data for the management of patients with tetanus-risk wounds is low and decreases with age, becoming minimal in geriatric patients. Elderly and women are less likely to have an effective vaccination status against tetanus.

## Introduction

### Background

Literature has only recently started considering the importance and relative unreliability of anamnestic data collected from patients presenting at emergency departments (EDs) (1–7).

Tetanus infection remains one of the most important possible complications of wounds. Since most guidelines for the treatment of this condition rely only on anamnestic data that are collected directly from the patient, the congruence between anamnesis and laboratory evidence must be verified. Most of the recently published studies on patient “reliability” were mainly focused on the anamnesis of psychiatric patients (8–10).

The reliability of anamnestic data is an even greater problem when we take into consideration the elderly population, in which the reliability of the anamnestic data seems to be lower (11). Focusing the analysis on elderly patients appears even more important if we consider that infections in the geriatric population are often overlooked in the early stages and that both infections and trauma have worse outcomes in the elderly (12–16).

However, work investigating the congruence between anamnestic data and laboratory test results should be extended and appears particularly important in the setting of vaccination (17). Tetanos Quick Stick (TQS) was one of the various tests introduced to assess the state of tetanus immunity. This is the most commonly used assay (tetanus enzyme-linked immunosorbent assay, ELISA) to detect anti-tetanus IgG antibodies. The performance of rapid tests for tetanus immunity is generally validated against ELISAs. The test is normally conducted in the laboratory rather than at the point of care because hospital Eds do not usually possess the specialist equipment required (5, 18, 19). The TQS test is an immunochromatographic test for the rapid detection of antitetanic antibodies in human serum, plasma, or whole blood (20). It is indicated for determination of the real immune status and the identification of unprotected individuals. Determining immune status also helps prevent against side effects in response to revaccination.

However, according to the World Health Organization (WHO) algorithm (21), the type of tetanus prophylaxis that is required following injury depends on the nature of the lesion and the history of previous immunizations. No booster is necessary if the last dose of the primary series or a subsequent booster injection was given <5 years ago for dirty wounds or <10 years ago for clean wounds.

Between 2001 and 2009 in Italy, a total of 594 tetanus cases were reported, with an average annual incidence of 1.0/1,000,000 population. The mean annual number of reported deaths was 21. Moreover, the incidence of clinical tetanus in Italy is 10-fold higher than that in other industrialized countries, likely due to higher susceptibility levels in Italy (12).

In 2010, Italy accounted for 57 of the 74 confirmed cases reported in the EU and has been continuously reporting the highest number of tetanus cases since 2006, ranging between 53 and 64 cases per year (22–32).

Although a more recent analysis from the European Centre for Disease Prevention and Control reported a decrease in incidence between 2010 and 2014, a further increase was observed in 2015, according to a WHO survey (11, 12, 33–36). This trend is likely a result of the introduction of the “universal” vaccination campaign for all infants, which has led to an 86% reduction between the mid-1950s and the present days. Today in Italy, tetanus affects only subjects who are either unvaccinated or inadequately vaccinated. Unfortunately, these subjects account for a sizable proportion of the general population.

These data underline how tetanus infection is still not as eradicated as believed and indicate that the population is not sufficiently aware of the possible complications of a simple wound.

Therefore, there is a need for interventions regarding the management of patients presenting with tetanus-prone wounds and the education of patients on how to be more responsible for their own health status. Our study aimed to assess the reliability of anamnestic data in the management of patients with tetanus-risk wounds, to identify categories of populations that are most exposed to non-vaccination coverage, and to assess the concordance of the TQS results to the therapy performed [administration of tetanus vaccine or immunoglobulin (IG)].

### Study Aim

#### Assess the Reliability of Anamnestic Data in the Management of Patients With Tetanus-Risk Wounds

The purpose of this study was to assess the reliability of anamnestic data in the management of geriatric patients (>65 years old) with tetanus-risk wounds at our ED. The secondary goals were to determine the reliability of anamnestic data in the overall population whether the reliability of the anamnestic data in the management of these patients differs depending on sex or age.

#### Assessment of the Study Population's Vaccination Status

We then assessed the vaccination status of the geriatric population (>65 years old) as determined by the TQS test. In particular, we looked at whether sex could provide more or less anti-tetanic coverage. Then we also analyzed the non-geriatric population as a comparison group and we have divided this population into three age categories (0–17, 18–44, 45–65) to determine whether any of these age groups were more exposed to the absence of coverage.

#### Assessment of Concordance of the TQS Result to the Therapy Performed (Administration of Tetanus Vaccine or IG)

This study was also performed to identify any possible margin of improvement that could be offered by the systematic use of the TQS test to determine the immune status of patients and to administer the best prophylaxis possible independently from the information gathered from questioning the patient. Therefore, we evaluated the concordance between the administration of the tetanus vaccine or IG and the TQS test results.

## Methods

### Overall Design

Eligibility criteria: Patients who accessed the ED of San Matteo Hospital Foundation, Pavia, Italy, for wounds between April 1, 2016, and December 31, 2017; whose state of consciousness was not altered; could read; and consented to the processing of data for health and research purposes.

The geriatric population is made up of patients over 65 years of age. The control group from patients under the age of 65. The exclusion criteria were severely bleeding wounds in need of immediate surgical intervention and the inability to provide a reliable history (i.e., psychiatric disease, dementia or confusion, patient in traumatic shock, unconsciousness).

The patients were asked whether they knew their immunization status against tetanus infection. The decision to administer a vaccine or IG was therefore clinical and based on anamnestic data, according to the WHO's position paper.

The TQS we used was Tetanos Quick Stick® distributed by InGenBioSciences.

The TQS test was given to patients who were unaware of their immunity status. Some patients who thought they knew their immunity but were not sure or in the clinical judgment of the traumatologist were given the TQS test to determine whether anamnestic collection was supported by the test.

These patients are represented by the following categories:

- Patients who think they know their vaccination status but do not declare themselves completely sure about the answer given;
- Patients not judged to be reliable by the traumatologist (poorly compliant, dementia, alcohol or drug abuse);
- Patients who expressed perplexity about what they had declared at the end of the visit and therefore requested the test.

The TQS results were then compared with the anamnestic data.

### Study Design

This was a retrospective single-center observational study with retrospective data collected through the software PiEsse. The agreement between the anamnestic patient-reported anti-tetanus coverage data and the TQS result was analyzed. Vaccine or IG administration (in line with the WHO position paper) and the TQS results were also analyzed.

Data were provided directly by the San Matteo Hospital Foundation, which keeps the files regarding all services that are provided by its ED. An *ad hoc* query was performed to obtain the data of interest. The names and surnames of the patients were substituted with an anonymous code to ensure that the researchers were blinded to the patient identities.

The data collection was retrospective; at the time of admission to the ED of the San Matteo Hospital Foundation, the patient provided informed consent for the processing of data for medical and research purposes. A register of Microsoft Excel was then utilized to collect all the data for subsequent epidemiological and statistical analyses.

### Statistical Analysis

Continuous variables were described by mean and 95% confidence interval, while qualitative variables were described through percentages.

Comparisons of continuous variables between the groups were performed with the Student *t*-test when appropriate, while associations between qualitative variables were studied with the χ^2^ test or Fisher's exact test when the number of observations within at least a single cell was equal to or <5. Concordance between self-declared anti-tetanus coverage and the TQS result has been tested by using the McNemar test. Appropriate logistic regression models were carried out to test the association between TQS test results and age and sex.

The significance level was set at alpha 0.05 (statistical significance at *p* < 0.05), and all tests were two-sided. The analyses were conducted with STATA software, version 14 (Stata Corporation, College Station, 2015, TX, USA).

### Tétanos Quick Stick

The Tétanos Quick Stick is a rapid test for assessing the state of immunity against tetanus. The test consists of a solid phase coated with tetanus toxoid and colloidal gold. The blood obtained by finger prick is applied to the well: if antibodies to tetanus are present in the sample, they form a complex with the conjugate of the solid phase and a pink stripe appears in the Test window. The pink stripe, which appears in window Control, is for quality control. It is a semiquantitative immunochromatographic test based on the ELISA method: the detection limits were tetanus antibody concentrations of 0.2 IU/ml in whole blood and 0.1 IU/ml in serum, thresholds below which the result of the test is negative. Positive TQS means protective immune status because the threshold is above the level of antibodies considered protective by the WHO, which is 0.01 IU/ml, using neutralization tests. A negative test shows, in most cases, a true level of <0.01 IU/ml of neutralizing antibodies, which is not protective.

## Results

### Assessment of the Reliability of Anamnestic Data in the Management of Geriatric Patients With Tetanus-Risk Wounds

The dataset contained 620 individuals (355 males and 265 females; 278 geriatric patients and 342 control group). The principle features of the patients included in the analysis, by sex, are shown in Table 1. The non-geriatric patients were then subdivided according to their age into three categories: 0–18, 19–45, and 46–65 years old. Of the 620 patients, 114 were not tested with the TQS. The study findings indicate that 424 out of 620 patients (68.38%) did not know their immune status.

**Table 1:** Principal features of patients included in the analysis, by sex.

	**Male *N* (%)**	**Female *N* (%)**	***p*** ***^a^***
**Age (years)**			
< 18	17 (4.79%)	8 (3.02%)	
18–44	120 (33.80%)	50 (18.87%)	
45–59	86 (24.23%)	61 (23.02%)	
60+	132 (37.18)	146 (55.09%)	<0.001
**Nationality**			
Italian	337 (94.93%)	249 (93.96%)	
Foreigner	18 (5.07%)	16 (6.04%)	0.601
**Reportedvaccination status**			
Yes	25 (7.04%)	10 (3.77%)	
No	111 (31.27%)	49 (18.49%)	
Unknown	219 (61.69%)	206 (77.74%)	<0.001
**TQS**			
Negative	212 (59.72%)	221 (83.40%)	
Positive	56 (15.77%)	17 (6.42%)	
Unknown	87 (24.51%)	27 (10.19%)	<0.001
**Immunoglobulinprophylaxis**			
Yes	225 (63.38%)	203 (76.60%)	
No	129 (36.34%)	60 (22.64%)	
Deny treatment	1 (0.28%)	2 (0.75%)	<0.001
**Tetanicvaccination**			
Yes	236 (66.48%)	214 (80.75%)	
No	117 (32.96%)	48 (18.11%)	
Deny treatment	–	2 (0.75%)	
Notavailable	2 (0.56%)	1 (0.38%)	<0.001
**Occupationalaccident**			
Yes	16 (4.51 %)	7 (2.64%)	
No	339 (95.49%)	258 (97.36)	0.224

a *Reported p-values are obtained by χ^2^ test*.

Among those who reported knowing their immune status, there was no agreement between the vaccine coverage declared by the patients and the TQS test results (*p* < 0.001). This figure was confirmed in both women (*p* < 0.001) and men (*p* = 0.041) (Table 2).

**Table 2:** Concordance between patient-declared vaccination coverage, specific immunoglobulins (IG) administration, and tetanic vaccination and the TQS test results.

	**TQS**		***p*** ***^a^***
	**Positive (%)**	**Negative (%)**	
**Declaredvaccinationcoverage**			
**Male**			
Yes	26 (63.4)	15 (36.6)	
No	5 (38.5)	8 (61.5)	*P* = 0.041
**Female**			
Yes	8 (27.6)	21 (72.4)	
No	0 (-)	7 (100)	*P* < 0.001
**Total**			
Yes	34 (48.6)	36 (51.4)	
No	5 (25.0)	15 (75.0)	*P* < 0.001
**IG administration**			
**Male**			
Yes	14 (7.1)	184 (92.9)	
No	42 (60.9)	27 (29.1)	*P* < 0.001
**Female**			
Yes	6 (3.1)	187 (96.9)	
No	11 (25.6)	32 (74.4)	*P* < 0.001
**Total**			
Yes	20 (5.1)	371 (94.9)	
No	53 (47.3)	59 (52.7)	*P* < 0.001
**Tetanicvaccination**			
**Male**			
Yes	16 (7.7)	191 (92.3)	
No	40 (67.8)	19 (32.2)	*P* < 0.001
**Female**			
Yes	8 (4.0)	194 (96.0)	
No	9 (26.5)	25 (73.5)	*P* < 0.001
**Total**			
Yes	24 (32.9)	385 (89.7)	
No	49 (67.1)	44 (10.3)	*P* < 0.001

a *Reported p-values are obtained by McNemar test*.

With regards to the agreement between patient-declared vaccination coverage and the TQS test results, we see that this association tends to decrease with increasing age, becoming maximum in geriatric patients.

### Assessment of the Study Population's Vaccination Status

In our study, the TQS results show that the proportion of the population that was effectively vaccinated by tetanus decreased with age, becoming minimal in geriatric patients (Table 3). The significance of this trend is confirmed in the logistic regression (Table 4). When evaluating the distribution of the TQS test results by sex, we see that women were significantly less likely to have positive TQS results than men, which is confirmed in the logistic regression (Table 4).

**Table 3:** Distribution of the TQS test results by age and sex.

	**TQS**			
	**Positive (%)**	**Negative (%)**	**Nottested (%)**	
**Age class**				
< 18	3 (12.0)	9 (36.0)	13 (52.0)	
18–44	25 (14.7)	105 (61.8)	40 (23.5)	
45–65	26 (17.7)	96 (65.3)	25 (17.0)	
65+	19 (6.8)	223 (80.2)	36 (13.0)	*P* < 0.001
**Sex**				
Male	56 (15.8)	212 (59.7)	87 (24.5)	
Female	17 (6.4)	221 (83.4)	27 (10.2)	*P* < 0.001

**Table 4:** Odds ratio (OR) of having a positive TQS by age class, age (continuous variable), and sex.

	**Odds ratio**	**95% Confidenceinterval**	***P***
Age class^*^			
<18	1 (reference)	–	–
18–44	0.32	0.13–0.78	0.012
45–65	0.31	0.12–0.76	0.011
65+	0.15	0.06–0.38	<0.001
Age^#^	0.98	0.97–0.99	<0.001
**Sex^§^**			
Male	1 (reference)	–	–
Female	0.34	0.19–0.62	<0.001

* *Adjusted for sex*.

# *Age as a continuous variable, adjusted for sex*.

§ *Adjusted for age*.

#### Assessment of the Concordance of the TQS Result to the Therapy Performed (Administration of Tetanus Vaccine or IG)

Our data show a statistically significant discrepancy between the therapies performed based on the anamnestic data and the TQS test results. This occurred both for the administration of a new tetanus vaccine (*p* < 0.001) and in relation to the administration of IG (*p* < 0.001) (Table 2).

## Discussion

Our findings show that anamnestic data are unreliable for detecting the effective immune status of geriatric patients. The study shows that this is also valid in the control group, in the various age groups, but reaches its maximum validity in the geriatric population. They also reveal how following an algorithm for administering antitetanic IG or tetanus vaccines can lead to inappropriate therapy administration or the non-administration of necessary therapy.

### Reliability of Anamnestic Data in the Management of Patients With Tetanus-Risk Wounds

The reliability of the anamnestic data is already greatly reduced by the high percentage of patients who do not remember their vaccination status in the ED setting. This could be due to the fact that patients do not pay attention to the importance of being immunized against some diseases and to the fact that it is extremely rare for patients who come to the ED to bring their vaccination card, from which it is possible to determine immune status. This fact is even more important in the geriatric population, in which the reliability of anamnestic data is a problem also for other pathologies and in ordinary contexts. It should be emphasized that this reliability is further reduced in the context of the emergency (1–7, 13, 37).

This is expected because emergency medicine is a challenging discipline that is characterized by a wide range of clinical conditions, the need for a high level of alacrity, and a substantial lack of clinical information on which to base the clinical approach to the patient. These factors contribute to making emergency medicine a high-risk discipline that is particularly prone to medical errors in both diagnosis and management (13). In emergency setting the unreliability of anamnestic data and clinical presentation may be also masked in the diagnosis of tetanus (38). In fact, it is known that the overcrowding of EDs correlates with adverse events, which is why various solutions have been proposed (39, 40).

However, the unreliability of anamnestic data is even more severely reduced by the demonstration, in our results, that too often there is no agreement between what is stated and the actual vaccination status of patients. It is important to underline that this, although more represented in the geriatric population, is also valid in the control population and in the various age groups. Other previous studies have also shown the discordance between the results of the TQS test and anamnesis regarding information on the immunization status (41–43). These mistakes in the knowledge of one's own immunization status lead to a risk of over-immunization, with its related possible complications (44–46). The most important risk is that constituted by patients who are in fact not immunized and who, when basing the therapeutic approach only on the anamnestic data provided by the patient, would not receive any type of prophylaxis. This could result in the subsequent development of an acute episode of tetanus, with the necessity of applying all the aforementioned measures (administration of antibiotics, benzodiazepines, mechanical ventilation, and supportive care) and therefore resulting in a prolonged hospital stay (47).

This analysis confirmed previous findings that anamnestic data on patients' immune status for tetanus are often unreliable (1–6, 14, 48–50).

### Study Population's Vaccination Status

The geriatric population in our study has the lowest immunity. Similar to that reported by other authors, the most important factor associated with a lower immunity rate was increased age (1, 51). This finding is likely due to a combination of the lack of systematic vaccination before 1962, increased life expectancy, the lack of administration of the recommended tetanus booster, the decrease in tetanus protective antibody levels as age increases, and a deficient immune response to vaccines associated with immunosenescence (32). Hammarlund et al. demonstrated the age-related decrease in IgG levels: seroprotection against tetanus without further booster vaccination was reliable for up to 72 years in 95% of the studied population (52). Also the longitudinal study of Amanna et al. confirmed seroprotection for 64 years in 95% of the subjects (53). The age-related immune senescence, in which antibody responses decay more rapidly with advanced age, explain therefore the lower antibody titers found in the elderly.

The data emerging from our study are in line with wider European studies: a study in Austria showed how the proportion of the population that was effectively vaccinated by tetanus decline with increasing time since the last vaccination. At all time-points antibody concentrations were lower in elderly people compared to young adults (51). This phenomenon agrees with what emerges from a study on the Israeli population (54). Another study shows that about 18% of adults above the age of 40 years, for whom the last vaccination dated back more than 20 years, were not protected against tetanus (55). An Austrian study of people aged more than 60 years undertaken by our group in 2005 revealed that 12% of the participants were not protected against tetanus (56).

These data confirm that the elderly are at increased risk because they are less covered by the vaccine, and we can therefore say that the analyzed group of patients is representative of the Italian reality as described by the Italian Ministry of Health (44). For the category of geriatric patients (older than 65 years), it is possible to denote a difference among males and females in immunization rates. In particular, a slightly higher percentage of male patients in this age group were protected against tetanus with respect to female patients. In the coming years in Italy, this finding could undergo a significant change, with a reduction in the difference between males and females aged 70 years or older.

As Filia et al. demonstrated, tetanus affected the female gender more frequently. Their serological data confirmed that elderlies (aged >65 years) were not protected against tetanus and underlined women were less likely to be protected than men. Inadequate vaccination coverage due to past vaccination strategies is probably the reason. The majority of people aged >65 years never received a primary vaccination series and women have less opportunities of being vaccinated during her lifetime than men, because they are exempted from military service. Furthermore, they had less probability to receive vaccination for work reasons (18).

This reduction may develop because the Military Service has not been obligatory since 2005 (57) and because of the obligatory vaccination schedule that was introduced in 1968 (58). Furthermore, more recent laws (59, 60) reaffirm that for individuals between 0 and 16 years old, a series of vaccinations is mandatory and administered without charge. Therefore, there will be a greater homogeneity between older males and females over time. However, based on this data analysis, when considering the number of patients in every age range who presented with wounds to the ED, even younger patients are lacking protective immunity.

It is essential to understand our results to know the history of the introduction of the tetanus vaccine in Italy. In Italy, tetanus toxoid vaccine was introduced in 1938 and was initially compulsory only for military personnel. In the early 1960s it became compulsory for 2-year-old children and for specific work categories and in 1968 for all newborns. Nowadays in Italy the vaccination schedule includes five doses of vaccine: three doses in the first year of life, followed by a booster dose at 5–6 years of age and one at 11–18 years. Additional booster doses are recommended every 10 years.

In Europe effective vaccination status varies greatly between the various states; generally, antibodies were higher in Austria, Belgium, and Germany than in Italy, Greece, and Poland. In most countries antibody concentrations decreased with increasing age. This occurrence is more evident in countries with generally lower antibody levels, such as Italy, Poland, and Greece (61).

The lack of protective immunity may be caused by the lack of knowledge about the importance of the prevention of this disease through a complete cycle of vaccinations and the lack of awareness on the necessity to receive boosters once the primary immunization series is completed. Tetanus is currently one of the most underestimated and less well-known possible complications of a wound, which leads to this lack of knowledge.

Since only three patients out of the 620 analyzed refused the vaccination in the ED, patients are most likely not immunized due to a simple lack of information on the importance of preventing *C. tetani* infection through a complete vaccination schedule. Patients should be reminded to request the subsequent boosters of the vaccine when necessary to receive the complete vaccination schedule. Many people may not be aware that tetanus-prone wounds require a booster to be given if more than five years have elapsed since the last immunization, as opposed to 10 years for low-risk wounds.

It has previously been demonstrated that among tetanus victims whose immunization histories were known, 72% had never completed a primary immunization series (62). It is extremely important to instruct patients on the topic of vaccines and vaccine preventable infections, stressing how some of these infections can quickly lead to major complications and eventually result in the death of the individual, and to teach patients how to be more responsible of their own health status.

### Possible Contribution of the TQS Result to the Therapy Performed

Despite this evidence, the identification of patients presenting with traumatic wounds and determining who will require the administration of either IG, the vaccine, or both is traditionally performed by collecting anamnestic data. However, the use of anamnestic data has been demonstrated to be a highly imprecise practice, particularly in ED settings, which are characterized by a chaotic and often emotionally charged environment (1–7, 14–16).

It therefore would be extremely important, time saving, and cost saving to acquire a more precise diagnostic tool that does not give excessive weight to the information reported by the patient.

Moreover, a subsequent algorithm based on the results of this diagnostic tool should be defined to allow the practitioners working in the ED to act in a safer and more appropriate way (3).

The rapid test (TQS) has been demonstrated to be a useful and reliable tool in the evaluation of tetanus immunity for patients with soft tissue injuries, as previously highlighted in the literature (5, 16, 63), because it is rapid, simple, diagnostic, and cost-effective, eliminates the unnecessary administration of vaccines, and may be performed in hospital settings (33). To this regard, Hamid Reza Hatamabadi revealed 88.1% sensitivity and 97.6% specificity for the TQS test. In particular, the positive and negative predictive values of TQS test were 99.3 and 66.1%, respectively. However, it also showed a significant decrease in cost when TQS was applied for patients with dirty, tetanus prone wounds or injuries and unknown or incomplete vaccination history (€ 9.48 vs. € 12.1) (49).

The diagnosis of protected wounds is often limited and can be improved by the use of these rapid diagnostic tests in the ED. More widespread use of this test would reduce the inappropriate prescription of IGs in protected patients and would limit the proportion of unprotected patients receiving no preventive treatment.

## Conclusion

The reliability of anamnestic data in the management of patients with tetanus-risk wounds is low and decreases with age, becoming minimal in the geriatric population. Trusting the anamnestic data could therefore lead to either lack of coverage of the infection or improper administration of vaccine and/or immunoglobulin. Instead, running a laboratory test could lead to averting these hypotheses. Elderly and women are less likely to have an effective vaccination status against tetanus. This calls for more caution for these sections of the population.

## Data Availability Statement

The raw data supporting the conclusions of this article will be made available by the authors, without undue reservation.

## Author Contributions

GS: concept and design. GS, IC, MB, AV, VF, and EO: involved in data analysis and interpretation. GS and EO: involved in drafting article. GS, IC, MB, IF, BC, GR, and MC: involved in critical revision and approval. EO: involved in statistics. All authors contributed to the article and approved the submitted version.

## Conflict of Interest

The authors declare that the research was conducted in the absence of any commercial or financial relationships that could be construed as a potential conflict of interest.

## Publisher's Note

All claims expressed in this article are solely those of the authors and do not necessarily represent those of their affiliated organizations, or those of the publisher, the editors and the reviewers. Any product that may be evaluated in this article, or claim that may be made by its manufacturer, is not guaranteed or endorsed by the publisher.
